# The Role of Clinical and Instrumented Outcome Measures in Balance Control of Individuals with Multiple Sclerosis

**DOI:** 10.1155/2013/190162

**Published:** 2013-05-25

**Authors:** Neeta Kanekar, Alexander S. Aruin

**Affiliations:** Department of Physical Therapy, University of Illinois at Chicago, 1919 West Taylor Street, Chicago, IL 60612, USA

## Abstract

*Purpose*. The aim of the study was to investigate differences in balance control between individuals with multiple sclerosis (MS) and healthy control subjects using clinical scales and instrumented measures of balance and determine relationships between balance measures, fatigue, and disability levels in individuals with MS with and without a history of falls. *Method*. Twelve individuals with MS and twelve healthy controls were evaluated using the Berg Balance and Activities-specific Balance Confidence Scales, Modified Clinical Test of Sensory Interaction on Balance, and Limits of Stability Tests as well as Fatigue Severity Scale and Barthel Index. *Results*. Mildly affected individuals with MS had significant balance performance deficits and poor balance confidence levels (*P* < 0.05). MS group had higher sway velocities and diminished stability limits (*P* < 0.05), significant sensory impairments, high fatigue and disability levels (*P* < 0.05). Sway velocity was a significant predictor of balance performance and the ability to move towards stability limits for the MS group. For the MS-fallers group, those with lower disability levels had faster movement velocities and better balance performance. *Conclusion*. Implementation of both clinical and instrumented tests of balance is important for the planning and evaluation of treatment outcomes in balance rehabilitation of people with MS.

## 1. Introduction

Multiple sclerosis (MS) is a chronic autoimmune demyelinating disease affecting the central nervous system [1]. Postural imbalance is often described as one of the initial symptoms of MS [2–5]. It is one of the most disabling MS symptoms that affects about 75% of patients during the course of the disease [6, 7]. People with MS also fall frequently [8–12], experiencing injurious falls [10, 13, 14]. Poor balance control is a significant contributing factor to the increased risk of falling in individuals with MS [2, 8–12, 15] and is also associated with lower engagement in physical activity [16]. Likewise, fear of falling is also associated with an increased risk of falls in MS [9, 17], and over 80% of people with fear also report activity curtailment [17]. Furthermore, people with MS identify fatigue as one of the primary reasons for falling [10, 18]. 

Balance control in people with MS has been commonly assessed through performance-based clinical measures such as the ability to maintain steady stance under conditions of reduced base of support with and without visual inputs [19–21], the ability to move towards stability limits [4, 20, 21], and through clinical measures or tests of balance performance such as the Berg Balance Scale [11, 22–25], Timed-Up and Go test [11, 24], and Activities-specific Balance Confidence scale [23, 24]. However, clinical measures of balance performance do not always characterize the underlying balance impairments. In the recent past, instrumented measures of balance such as center of pressure excursions and sway analysis [12, 22, 25–28] and computerized dynamic posturography [5, 15, 29] have been used to quantify balance impairments in people with MS. These measures are considered to be more sensitive than common clinical tests for documenting balance deficits and provide a reliable tool to identify subjects at risk of falls, even in minimally impaired people with MS [25]. As such, it is important to investigate the relationships between the clinical and instrumented outcome measures. An insight into the associations between various balance measures would increase the understanding of the specific impairments that may be targeted during balance rehabilitation in people with MS. To this end, the first objective of this study was to determine differences in balance control between individuals with MS and healthy control subjects based on clinical scales and instrumented measures of balance. The second aim was to determine the association between different measures of balance control, fatigue, independence levels, and disability levels in people with MS and to investigate the underlying causes of balance deficits in MS. The study was also aimed at exploring the differences and relationships between various clinical and instrumented outcome measures in people with MS with and without a history of falls.

## 2. Methods

Twelve individuals with relapsing-remitting MS and twelve age- and gender-matched healthy control (HC) subjects participated in the study (Table 1). The MS group had an EDSS [30] score of 2.3 ± 0.9, indicating mild disability and fully ambulatory status. The inclusion criteria for the individuals with MS were normal or corrected to normal vision and the ability to stand independently without any aid or orthosis for at least one minute. The study was approved by the university's Institutional Review Board.

Both the groups of subjects (MS and HC) were assessed on two clinical balance scales (performance measures) and on two instrumented balance tests (impairment measures) as well as for fatigue level, foot sensation, and independence in activities of daily living. All subjects were tested bare foot in one session, and subjects were allowed to rest during testing if necessary. Individuals with MS were tested in the morning to minimize the effects of fatigue commonly reported later in the day. The MS group also reported the number of falls during the past six months: those who had two or more falls were classified as fallers (MS-F) and those who had less than two falls were classified as nonfallers (MS-NF) [8]. 

### 2.1. Clinical Balance Measures

Berg Balance Scale (BBS) and Activities-specific Balance Confidence (ABC) scale were implemented. BBS assesses static balance using 14 items with a maximum total score of 56 [31]. The BBS has been reported as a valid [24] and reliable [23] tool for the population with MS. ABC scale is a 16-item self-report questionnaire rating balance confidence in performance of various ambulatory activities without losing balance with scores ranging from 0%—no confidence—to 100%—completely confident [32]. The ABC scale has been reported as a valid [24] and reliable [23] scale on balance confidence in people with MS.

### 2.2. Instrumented Balance Measures

Modified Clinical Test of Sensory Interaction on Balance (mCTSIB) and Limits of Stability test (LOS) were implemented using the Balance Master protocol (NeuroCom, USA). The mCTSIB assesses sensory impairment or dysfunction by measuring the subject's center of gravity (COG) sway velocity during standing under four sensory conditions: eyes open firm surface (firm-eo), eyes closed firm surface (firm-ec), eyes open unstable surface (foam-eo), and eyes closed unstable surface (foam-ec). The mCTSIB examines how well an individual is using sensory inputs in maintaining balance when one or more sensory systems are compromised. The LOS test assesses impairment of voluntary motor function by quantifying the subjects' ability to move their COG (lean their body) to their stability limits in eight different directions without losing balance. The test measures movement reaction time (RT), movement velocity (MVL), endpoint excursion (EPE), maximum excursion (MXE), and movement directional control (DCL) for four main directions (anterior, posterior, right, and left) during an eight-second trial. A composite score is also calculated for each of the five variables. The ability to voluntarily move the COG to positions within the LOS is fundamental to mobility tasks such as reaching for objects, transitioning from a seated to standing position (or standing to seated), and walking.

### 2.3. Assessment of Fatigue, Sensations, and Functional Independence

Fatigue was assessed using the Fatigue Severity Scale (FSS), a 9-item self-report questionnaire rating the severity of fatigue symptoms, based on how accurately each statement reflects the individual's condition over the preceding week with scores ranging from 1 to 7, a higher score indicating more severe fatigue [33]. The FSS is found to be a reliable measure of fatigue severity in people with MS [34]. Sensory assessments included the use of the Semmes-Weinstein aesthesiometry set [35] to determine loss of pressure sensitivity (protective sensation) over the plantar surface of the foot and a 128 Hz tuning fork to assess vibratory sensation over the hallux of each foot. Sensory assessments were performed as per the Clinical Skills Module of the American College of Physicians. A person's level of independence in performing activities of daily living (ADL) was assessed using the Barthel Index (BI) on a 0–20 scale, with 0 indicating minimum independence and 20 representing maximum independence or least disability [36]. The BI is found to be a valid and reliable tool and is advocated as a standard measure of physical disability in MS and other neurological diseases [37, 38].

### 2.4. Statistical Analysis

All data were analyzed using SPSS for Windows, release 19.0, and are expressed as mean ± SD. Data normality was tested using Shapiro-Wilk test. To compare the differences between the MS and HC groups and between the MS-F and MS-NF groups, independent *t*-tests were performed for subject demographics, BBS, ABC Scale, composite scores on the mCTSIB and LOS tests, FSS, Sensory assessments, and the BI. For data not normally distributed, a nonparametric analysis using the Mann-Whitney *U* test was performed. Additionally, split-plot ANOVAs were performed to compare between group differences and interactions for the different conditions of the mCTSIB and LOS tests. The data were checked for sphericity and wherever the assumption of sphericity, was not met the Huynh-Feldt test was reported. A post hoc comparison was done using Tukey's Honestly Significant Difference test for significant interactions and main effects. Further analysis was performed on the MS group to determine the association between different clinical and instrumented scores of balance, FSS, and BI using the Pearson and Spearman's rank correlation coefficients. Regression analysis was run where the associations were found to be significant. Statistical significance was set at *P* < 0.05.

## 3. Results

### 3.1. Differences between MS and HC Groups

There were no significant differences in age, height, and weight between the MS and HC groups (Table 1).

#### 3.1.1. Clinical Balance Measures

**Figure 1:**
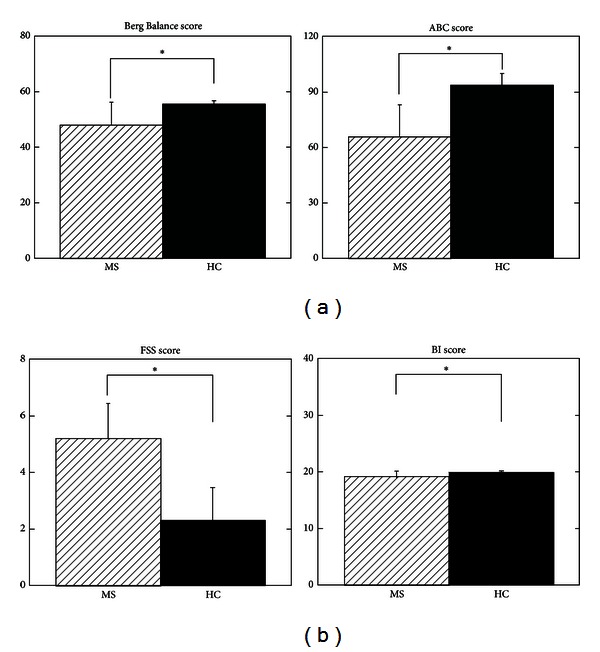
(a) Berg Balance score and Activities-specific Balance Confidence (ABC) scores for the group of individuals with multiple sclerosis (MS) and healthy control (HC) subjects. (b) Fatigue Severity Scale (FSS) and Barthel Index (BI) scores are shown for the MS and healthy control groups. ∗ denotes statistically significant differences at *P* < 0.05.

Balance performance of the MS group was significantly worse than the HC group on the BBS (*P* < 0.0001). The average score for the MS group was 47.91 ± 8.25 and for the HC group was 55.58 ± 1.16 (Figure 1(a)). The MS group reported significantly lower levels of balance confidence in performing various ambulatory activities than the HC group (*P* < 0.0001). The average ABC score for the MS group was 65.76 ± 17.33% and for the HC group was 93.85 ± 6.33% (Figure 1(a)).

#### 3.1.2. Instrumented Balance Measures

**Figure 2:**
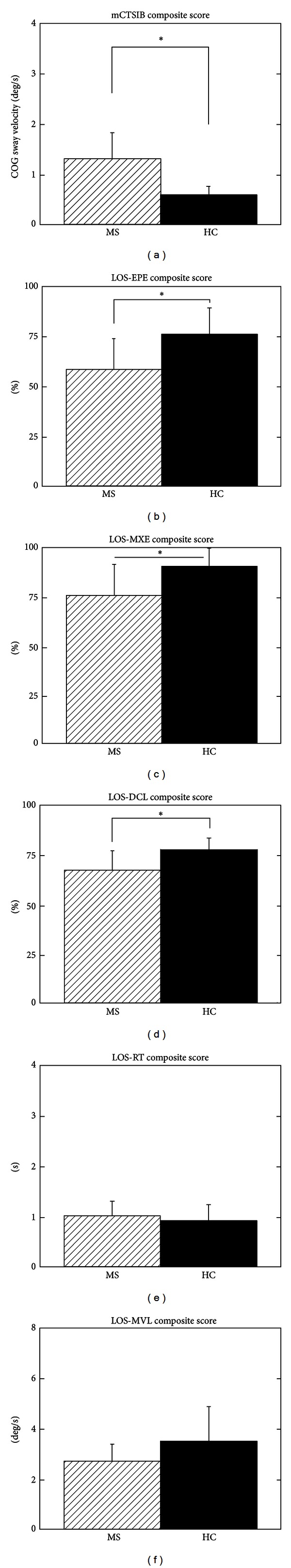
Modified Clinical Test of Sensory Interaction on Balance (mCTSIB) and Limits of Stability (LOS) test scores for the group of individuals with MS and healthy control (HC) subjects. COG: center of gravity, EPE: endpoint excursion, MXE: maximum excursion, DCL: movement directional control, RT: movement reaction time, MVL: movement velocity. ∗ denotes statistically significant differences at *P* < 0.05.

The mCTSIB test revealed that the composite COG sway velocity was significantly higher for the MS group (1.34 ± 0.49 deg/s) than the HC group (0.62 ± 0.15 deg/s) (*P* < 0.0001) (Figure 2). Moreover, ANOVA showed a significant group *x* condition interaction effect (*F*_(3,57)_ = 4.83, *P* = 0.032), a significant main effect of the group (*F*_(1,19)_ = 16.33, *P* = 0.001), and a significant main effect of the condition (*F*_(3,57)_ = 49.60, *P* < 0.0001). Posthoc test demonstrated that for each sensory condition, the MS group had higher COG sway velocities than the HC group. Posthoc test for the condition effect showed that for both groups, COG sway velocity increased in more challenging conditions; specifically the sway velocity was higher for the foam-ec condition as compared to the firm-eo (*P* < 0.0001), firm-ec (*P* < 0.0001), and foam-eo conditions (*P* = 0.003).

The composite scores for the LOS measures are shown in Figure 2. Both, the distance of the first movement towards the target (EPE) and the maximum distance reached (MXE) by the MS group, were significantly smaller than the distance reached by the HC group (MS: EPE was 58.45 ± 15.73% and MXE was 76.27 ± 14.92%, HC: EPE was 76.33 ± 12.67% and MXE was 90.33 ± 8.54%; *P* = 0.007 for EPE and *P* = 0.015 for MXE). Directional control (DCL) was also reduced in MS as compared to the healthy subjects (MS: 68.09 ± 9.21%, HC: 78 ± 5.46%; *P* = 0.005). Although the MS group had longer reaction times and lower movement velocities as compared to the HC group, it did not reach statistical significance. ANOVA showed a near significant group *x* direction interaction effect for MXE (*F*_(3,63)_ = 2.70, *P* = 0.05), a significant main effect of the group (*F*_(1,21)_ = 7.52, *P* = 0.012), and a significant main effect of the condition (*F*_(3,63)_ = 34.26, *P* < 0.0001). Posthoc test demonstrated that for each direction, the MS group had smaller MXE than the HC group. Specifically, the MXE for the MS group towards the right side was smaller as compared to the MXE for the HC group towards the right side (*P* = 0.037) and towards the left side (*P* = 0.038). Moreover, irrespective of the direction, the MS group had smaller MXE than the HC group (*P* = 0.012). 

#### 3.1.3. Fatigue, Sensory, and Functional Independence Measures

The MS group reported higher levels of fatigue than the HC group (*P* < 0.0001). The average FSS score for the MS group was 5.19 ± 1.25 and for the HC group was 2.3 ± 1.15 (Figure 1(b)). There was a significant group difference on the monofilament test, with 6 individuals of the MS group showing a loss of pressure sensitivity as compared to its presence in all subjects of the HC group (*P* = 0.008). There was no difference between the groups for the vibratory sensation test (*P* = 0.36). The scores on the BI for the MS group were significantly lower than the HC group (*P* = 0.01). The average score for the MS group was 19.16 ± 0.93 and for the HC group was 19.91 ± 0.28 (Figure 1(b)).

### 3.2. Relationship between Outcome Measures for the MS Group

**Figure 3:**
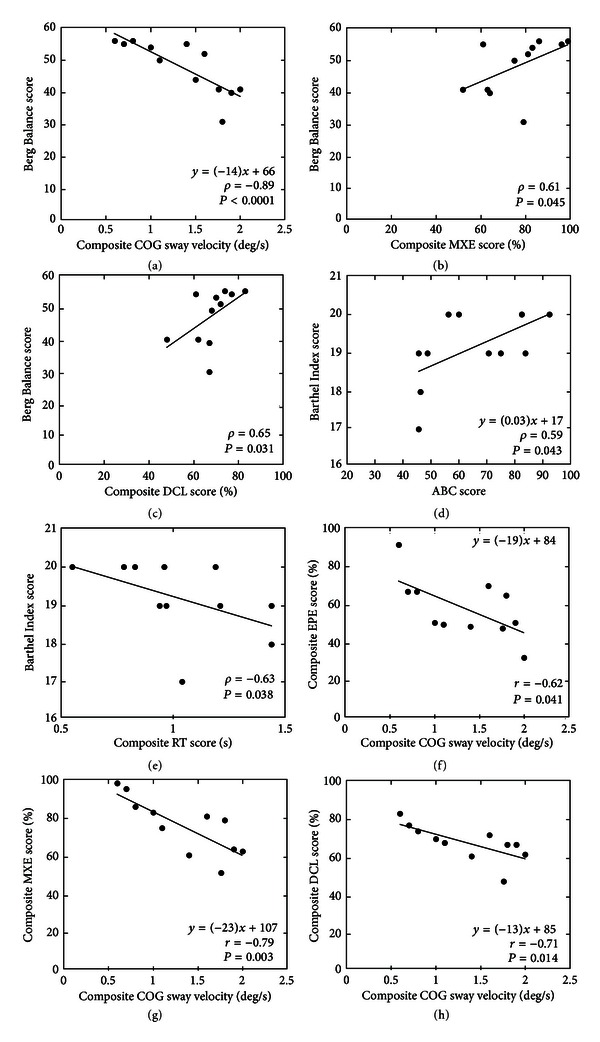
The results of correlation and regression analysis between clinical and instrumented outcome measures for the MS group. Abbreviations as defined in Figures 1 and 2 captions. All correlations and regression analyses shown are statistically significant (*P* < 0.05).

The results of correlation and regression analyses are shown in Figure 3. There was a significant negative correlation between composite COG sway velocity on the mCTSIB test and the BBS score (*ρ* = −0.89, *P* < 0.0001) and between sway velocity for the foam-eo condition and the BBS score (*ρ* = −0.72, *P* = 0.012). A significant positive correlation occurred between the composite MXE score and the BBS score (*ρ* = 0.61, *P* = 0.045) and between the composite DCL score and the BBS score (*ρ* = 0.65, *P* = 0.031). Multiple regression analysis was used to test if the composite COG sway velocity and the composite MXE and DCL scores significantly predicted the BBS scores for the MS group. The regression model explained 66.3% of the variance (*R*_adj_^2^ = 0.663, *F*_(3,7)_ = 7.55, and *P* = 0.013). It was found that composite COG sway velocity significantly predicted Berg balance score (*β* = −1.17, *P* = 0.007). The BI scores showed a significant positive correlation with the ABC score (*ρ* = 0.59, *P* = 0.043) and a significant negative correlation with the composite RT score on the LOS test (*ρ* = −0.63, *P* = 0.038). Regression analysis was used to test if the ABC score significantly predicted the BI score for the MS group. The regression model explained 27.7% of the variance (*R*_adj_^2^ = 0.277, *F*_(1,10)_ = 5.20, and *P* = 0.046) and the ABC score significantly predicted the BI score (*β* = 0.585, *P* = 0.046). Assessment of the relationship between the mCTSIB and LOS measures showed significant negative correlations of the composite sway velocity with the composite EPE score (*r* = −0.62, *P* = 0.041), with the composite MXE score (*r* = −0.79, *P* = 0.003), and with the composite DCL score (*r* = −0.71, *P* = 0.014). Regression analysis showed that composite sway velocity explained 32% of the variance (*R*_adj_^2^ = 0.32, *F*_(1,9)_ = 5.70, *P* = 0.041) in EPE and was a significant predictor of the EPE score (*β* = −0.623, *P* = 0.041) and it also explained 59.4% of the variance (*R*_adj_^2^ = 0.594, *F*_(1,9)_ = 15.61, and *P* = 0.003) in MXE and was a significant predictor of the MXE score (*β* = −0.796, *P* = 0.003). Furthermore, composite sway velocity explained 44.9% of the variance (*R*_adj_^2^ = 0.449, *F*_(1,9)_ = 9.133, and *P* = 0.014) in DCL and was a significant predictor of the DCL score (*β* = −0.71, *P* = 0.014). 

### 3.3. Differences between MS-F and MS-NF Groups

Six subjects in the MS group reported an average of 3 ± 0.89 falls (MS-F) and six subjects reported no falls (MS-NF). Subject demographics, BBS and ABC scores, and the mCTSIB and LOS measures were not significantly different between the two groups (*P* > 0.05, Tables 1 and 2). 

The MS-F group reported significantly lower levels of fatigue than the MS-NF group (*P* = 0.006). The average FSS score for the MS-F group was 4.31 ± 1.02 and for the MS-NF group was 6.08 ± 0.71 (Table 2). There was a loss of pressure sensitivity in 4 subjects of the MS-F group and in 2 subjects of the MS-NF group, whereas vibratory sensation was absent in only 1 individual of the MS-F group and it was intact in all subjects of the MS-NF group; the group differences were not statistically significant. The scores on the BI were not statistically different between the two groups (Table 2).

### 3.4. Relationship between Outcome Measures for the MS-F Group

**Figure 4:**
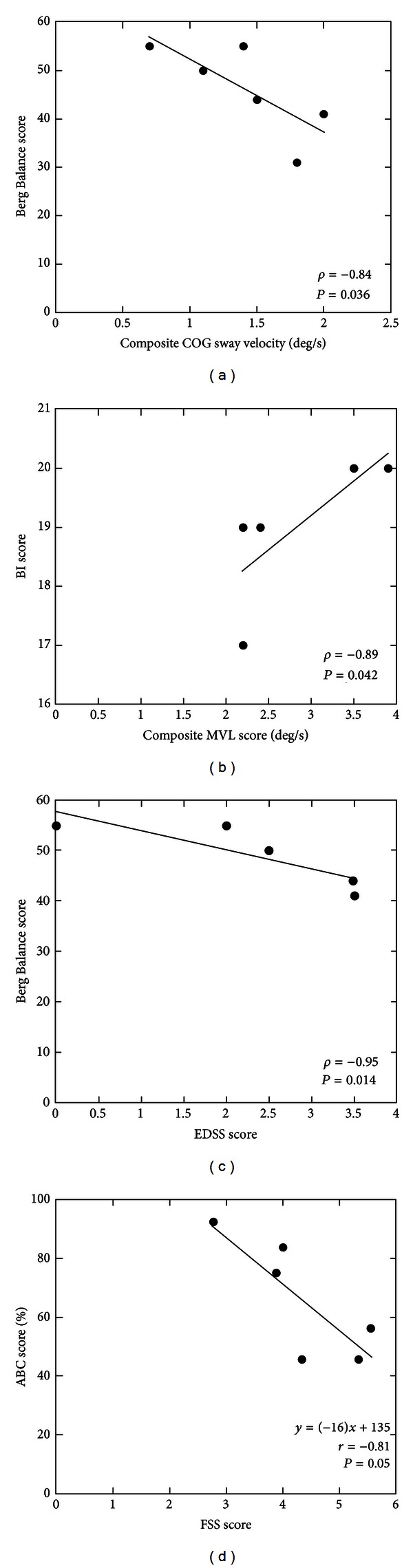
The results of correlation and regression analysis between clinical and instrumented outcome measures for the individuals with MS with a history of falls (MS-F). EDSS: Expanded Disability Status Scale. Other abbreviations as defined in Figures 1 and 2 captions. All correlations and regression analyses shown are statistically significant (*P* < 0.05).

The results of correlation and regression analyses are shown in Figure 4. There was a significant negative correlation between composite COG sway velocity on the mCTSIB test and the BBS score for the MS-F group (*ρ* = −0.84, *P* = 0.036). The scores on the BI for the MS-F group showed a significant positive correlation with the composite MVL scores on the LOS test (*ρ* = 0.892, *P* = 0.042). Furthermore, the level of disability on the EDSS score correlated negatively with the BBS score (*ρ* = −0.95, *P* = 0.014). The score on the ABC scale also showed a near significant negative correlation with the FSS score for the MS-F group (*r* = −0.81, *P* = 0.05). Regression analysis was used to test if the FSS score significantly predicted the ABC score for the MS-F group. The regression model explained 57.2% of the variance (*R*_adj_^2^ = 0.572, *F*_(1,4)_ = 7.68, and *P* = 0.05), and the FSS score significantly predicted the ABC score (*β* = −0.811, *P* = 0.05). 

## 4. Discussion

Deficits in balance control are a common and often an initial disabling symptom in MS [2, 3, 5]. Postural instability has been reported in mildly impaired people with MS [2, 4, 5] and even in those with normal clinical balance tests but subjective symptoms of balance impairment [3]. Several studies have highlighted the incidence of balance disorders in individuals with MS based on results of clinical balance and mobility tests. Our results also showed that people with MS had poor balance ability in maintaining and changing positions during standing and seated tasks despite low levels of disability on the EDSS scale. In addition, they also reported significantly lower levels of balance confidence on the ABC scale, similar to previous research findings [23, 24]. Balance impairments in MS have also been quantified through the use of force platform-based center of pressure and COG measures allowing instrumented assessment of clinically significant tasks. The findings of the mCTSIB test demonstrated that in general, people with MS swayed more than their healthy counterparts under all sensory conditions, indicating the presence of an impairment of sensory reweighing strategies. Similar results have been shown by several researchers [3, 15, 22, 28] signifying an increased level of noise and lower levels of accuracy in the sensory system of people with MS, thereby resulting in impaired weighing of inputs and consequential inaccuracy in the execution of appropriate motor commands to maintain postural stability [22]. In our study, a significant loss of pressure sensitivity of the foot sole was also observed which may have contributed to the increased sway velocities in MS. It has been recently shown that sensitivity of the cutaneous receptors of the foot sole is related to and predicts static standing balance in mild to moderately disabled persons with MS [19]. Our results of the LOS test confirmed with previous literature, findings demonstrating the diminished ability of people with MS in voluntarily reaching and leaning towards their limits of stability [4, 26, 28]. The MS group showed significantly smaller postural shifts while leaning towards their stability limits in all directions. For the first time, we found that people with MS had poor directional control as compared to the healthy counterparts probably due to the underlying demyelination and destruction in the cerebellar regions in MS [27]. Impairment in the ability to voluntarily move the COG towards stability limits can cause instability during activities of daily living such as reaching for objects [20, 21] and walking [39].

 The MS group also reported higher levels of fatigue than the healthy controls. Indeed, fatigue is one of the most common symptoms of MS, occurring in about 80% of people, and may be the most prominent symptom in people who otherwise have minimal activity limitations [10, 18], significantly interfering with a person's quality of life [40, 41]. Interestingly, while the MS group had mild disability on the EDSS scale, their score on the BI (while being relatively high) was significantly lower than the healthy subjects. Particularly, items related to bowel and bladder incontinence were commonly reported activity limitations, which are known to compromise unidimensionality within the construct of ADL [37]. 

### 4.1. Relationship between Clinical and Instrumented Measures of Balance, Fatigue, and Disability Levels in MS

The correlation analysis showed that people with MS with poor balance performance had higher COG sway velocities, especially in the foam-eo condition where somatosensory inputs are compromised. The composite COG sway velocity was found to be a significant independent predictor of balance performance on the BBS in MS. Likewise, higher scores on the BBS for people with MS were associated with larger maximum excursions and greater directional control of the COG on the LOS test; however, they did not predict performance on the BBS. The substantial relationships between these clinical and instrumented measures can be explained by the fact that these tests assess certain similar aspects of balance control. Additionally, people with MS with higher composite sway velocities had lower COG excursions and poorer directional control while leaning in different directions. The ability to maintain steadiness in standing was also found to be a significant independent predictor of the ability to move the COG towards stability limits. Such a causal relationship highlights the probable factors that may underlie the known impairment of mobility tasks such as gait initiation [39], walking (shorter strides and lower speeds) [4, 11, 42], and repeated stepping [20, 21] in people with MS. Additionally, people with MS with higher levels of balance confidence and faster reaction times were found to be more functionally independent in performing ADLs. In fact, balance confidence in ambulatory activities significantly predicted functional independence on the BI for the MS group.

### 4.2. Falls in MS

It has been reported that fall tendency may occur early in the disease course of MS, even before impairment of locomotion, and balance becomes evident on clinical examination [43]. In the current study, 50% of people with MS reported having fallen twice or more in the previous six months, although the disability levels were minimal and similar between fallers and nonfallers. Incipient balance and gait impairments are detected early on in MS when disability levels are mild [4, 42] and are known to be significant contributors of falls in people with MS [11, 15, 25, 27]. In the current study, however, while the fallers seemed to have balance deficits, the differences between the fallers and nonfallers were not statistically significant, probably due to small sample sizes and variability among subjects. Previous research has identified the occurrence of falls to be more frequent during the afternoon and when fatigued [10, 18]. Interestingly, the group of fallers in this study reported notably lower levels of fatigue than the nonfallers. A possible explanation for the increased number of falls in the MS-F group in spite of their lower fatigue levels may be due to the finding of an inverse relationship between fatigue and balance confidence levels in this group. People with lower levels of fatigue in the MS-F group had higher balance confidence levels; in fact the FSS score was a significant predictor of the ABC score. The subjects in the MS-F group may therefore have been likely to participate more often in ambulatory activities, thereby increasing their chances of experiencing a fall. Transfers and ambulation-related tasks are indeed the most commonly reported activities being performed at the time of a fall in people with MS [10]. This finding, however, should be confirmed in future studies where subjects should be asked to specifically report their mobility levels and the particular activities during which falls occurred. Analysis of the relationships between different outcome measures in the MS-F group showed that people with MS with poor balance performance on the BBS had higher COG sway velocities, especially with eyes closed. It was also found that people with low EDSS scores demonstrated better balance performance on the BBS; however, this relationship was not causal. These outcomes are in line with previous research that has found advancing disease status, greater postural sway velocity with eyes closed, and visually dependent sway as predictors of future falls in people with MS [11, 15, 25, 27]. 

In conclusion, the role played by both the clinical and instrumented outcome measures is significant, and a combination of tests could facilitate the characterization of the underlying balance impairments and thus provide a stronger foundation for the planning and evaluation of treatment outcomes in the rehabilitation of people with MS.

## Figures and Tables

**Table 1:** Demographics for the MS and HC groups.

Characteristics	MS group (*n* = 12, 10 F, 2 M)	HC group (*n* = 12, 9 F, 3 M)	*P* value	MS-F (*n* = 6, 5 F, 1 M)	MS-NF (*n* = 6, 5 F, 1 M)	*P* value
Age (years)	53.16 ± 13.38	51.83 ± 13.60	0.81	55.5 ± 15.37	50.83 ± 12.02	0.57
Height (cm)	170.25 ± 10.6	166.66 ± 9.28	0.38	169.33 ± 12.44	171.19 ± 9.52	0.77
Weight (kg)	68.68 ± 11.22	73.17 ± 11.35	0.33	69.70 ± 6.25	67.66 ± 15.34	0.77
EDSS score	2.3 ± 0.9	—	—	2.3 ± 1.44	2.17 ± 0.92	0.86
MS duration (years)	17.24 ± 11.05	—	—	18.83 ± 8.18	14.77 ± 13.13	0.53

F: female; M: male; mean ± standard deviations are shown.

**Table 2:** Test scores for the MS-F and MS-NF groups.

Outcome measure	MS-F (*n* = 6)	MS-NF (*n* = 6)	*P* value
BBS	46 ± 9.29	49.83 ± 7.38	0.44
ABC scale (%)	66.43 ± 20.08	65.10 ± 16.02	0.90
Composite COG sway velocity (deg/s)	1.41 ± 0.47	1.27 ± 0.54	0.64
Composite RT (s)	0.95 ± 0.33	1.10 ± 0.22	0.39
Composite MVL (deg/s)	2.84 ± 0.80	2.73 ± 0.56	0.81
Composite EPE (%)	52.80 ± 13.82	63.17 ± 16.84	0.30
Composite MXE (%)	74.80 ± 14.11	77.50 ± 16.79	0.78
Composite DCL (%)	67 ± 6.36	69 ± 11.62	0.74
FSS	4.31 ± 1.02	6.08 ± 0.71	0.006*
Protective sensation	Absent in 4	Absent in 2	0.27
Vibration	Absent in 1	Present in all	0.32
BI	19 ± 1.09	19.33 ± 0.81	0.60

Mean ± standard deviations are shown, *significant at *P* < 0.01.
